# Antibiotic Escalation Following Rapid Multiplex PCR Pneumonia Panel Testing in Intensive Care Patients with Severe Pneumonia: A Retrospective Cohort Study

**DOI:** 10.3390/antibiotics15090903

**Published:** 2026-09-14

**Authors:** Soo Kyun Jung, Keum Ju Choi, Eun Jin Kim

**Affiliations:** Division of Pulmonary and Critical Care Medicine, Department of Internal Medicine, Daegu Catholic University Hospital, Daegu Catholic University School of Medicine, Daegu 42472, Republic of Korea; jsk8585@cu.ac.kr (S.K.J.); tvbogo@cu.ac.kr (K.J.C.)

**Keywords:** rapid multiplex PCR, FilmArray pneumonia panel, severe community-acquired pneumonia, antibiotic escalation, antimicrobial stewardship, intensive care

## Abstract

Background/Objectives: Severe community-acquired pneumonia (CAP) can be fatal and requires the rapid and appropriate administration of antibiotics. Rapid multiplex PCR pneumonia panels are widely used tools for antimicrobial de-escalation and stewardship; however, their role in driving antibiotic escalation in severe pneumonia requiring intensive care is less well characterized. Methods: We retrospectively analyzed 288 adults with severe CAP who were admitted to the intensive care unit (ICU) of a tertiary hospital and underwent lower-respiratory BioFire FilmArray Pneumonia Panel (BFPP) testing between April 2023 and September 2024. The primary outcome was antibiotic escalation, defined as addition of an agent or a change to broader-spectrum therapy after the BFPP result, and its associated factors. Secondary outcomes were in-hospital mortality, hospital and ICU length of stay (LOS), and ventilator duration according to the antibiotic escalation. Results: Patients were elderly (median 72 years old; 60.8% male) and critically ill (median SOFA 8; median APACHE-II 19; shock 49.0%; mechanical ventilation 56.9%). The panel was positive in 66.3% of cases, and results were obtained with a median turnaround time of 2 h versus 67 h for sputum culture. After the panel, antibiotics were escalated in 34.0% of cases, de-escalated in 2.8%, discontinued in 0.7%, and unchanged in 62.5%. Escalation was independently associated with resistance gene detection (adjusted odds ratio [aOR] 3.15, 95% CI 1.71–5.82), panel positivity (aOR 1.97, 95% CI 1.02–3.80), and SOFA score (aOR 1.10 per point, 95% CI 1.01–1.19). Thirty-day mortality, in-hospital mortality, hospital LOS, ICU LOS, and ventilator duration did not differ between escalation and non-escalation groups. Conclusions: In severe CAP requiring intensive care and managed with a comparatively narrow initial empirical regimen, panel testing was predominantly followed by antibiotic escalation rather than de-escalation, particularly when resistance genes were detected. No statistically measurable difference in clinical outcomes was observed, but the study was not powered to exclude any clinically meaningful benefit or harm of escalation.

## 1. Introduction

Pneumonia is the leading infectious cause of death globally, and severe pneumonia represents the most critical spectrum of the disease. Severe pneumonia is frequently associated with intensive care unit (ICU) admission, respiratory failure when using ventilator therapy, septic shock, and death [1,2].

Administering early and appropriate antibiotic treatment can reduce both mortality and antimicrobial resistance rates; however, since the causative organisms and antibiotic resistance status of pneumonia are unknown, clinicians have little choice but to use broad-spectrum antibiotics. Thus, after starting broad-spectrum antibiotic treatment, it is necessary to switch to an appropriate range of antibiotics as soon as possible [3].

Conventional culture of respiratory samples is the diagnostic standard for pneumonia. However, this method has disadvantages, such as a lengthy turnaround time for results, reduced sensitivity following prior antibiotic exposure, and an inability to identify viral or atypical pathogens [4,5].

Rapid multiplex polymerase chain reaction (rm-PCR) assays have emerged as a solution to these issues. One such assay, the BioFire FilmArray Pneumonia Panel (BFPP; BioFire Diagnostics, bioMérieux), can identify the causative organism within 1–2 h and offers a sensitivity of 96.3% and a specificity of 97.2% for sputum and endotracheal aspirates, and a sensitivity of 96.2% and a specificity of 98.3% for bronchoalveolar lavage (BAL) samples [6]. Furthermore, this panel can report the antimicrobial resistance-related genes for methicillin-resistant *Staphylococcus aureus* (MRSA), extended-spectrum beta-lactamase (ESBL)-producing organisms, and carbapenem-resistant *Enterobacteriaceae* [7], which is expected to support rapid antibiotic selection. Therefore, the BFPP may facilitate timely and appropriate escalation of antimicrobial therapy in this high-risk population of multidrug-resistant (MDR) organisms.

This retrospective single-center study was conducted at a tertiary referral center with high institutional multidrug resistance prevalence that receives a substantial proportion of patients transferred from long-term care facilities and surrounding hospitals. We analyzed both the BFPP and conventional culture of respiratory samples in patients admitted to the ICU with severe pneumonia.

## 2. Results

### 2.1. Cohort Characteristics

Among the 288 patients, the median age was 72 years old and 60.8% were male. Illness severity was high (median SOFA 8, median APACHE-II 19), with shock in 49.0% and mechanical ventilation in 56.9% of cases (Table 1; Three groups are shown in Supplementary Table S1).

### 2.2. Panel Performance

The panel was positive in 191/288 (66.3%) of the patients. Median panel turnaround was 2 h, compared with 67 h for sputum culture (Table 1).

### 2.3. Primary Outcome

**Figure 1:**
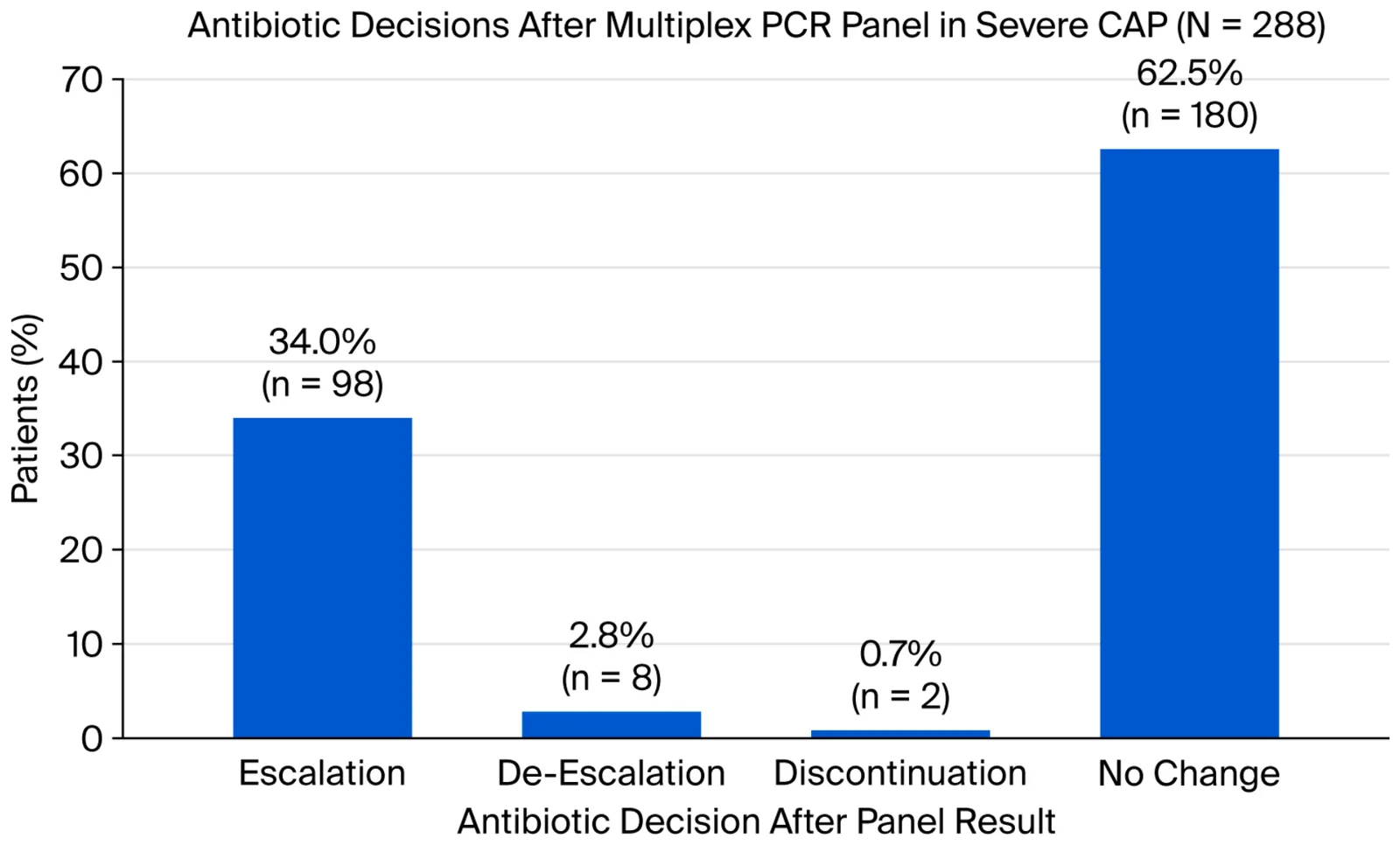
Distribution of antibiotic decisions after the multiplex PCR panel result in 288 patients with severe community-acquired pneumonia: escalation in 34.0% (n = 98), de-escalation in 2.8% (n = 8), discontinuation in 0.7% (n = 2), and no change in 62.5% (n = 180).

After the panel result, antibiotics were escalated in 98/288 cases (34.0%), de-escalated in 8 (2.8%), discontinued in 2 (0.7%), and unchanged in 180 (62.5%) (Figure 1).

### 2.4. Factors Associated with Escalation

Escalation occurred in 41.9% (80/191) of panel-positive patients versus 18.6% (18/97) of panel-negative patients (*p* < 0.001), and in 57.1% (48/84) of patients with a detected resistance gene versus 24.5% (50/204) without (*p* < 0.001) (Table 2).

Among the 84 resistance gene-positive results, methicillin resistance markers (mecA/mecC/MREJ) were detected in 36 cases (42.9%; 12.5% of the full cohort), CTX-M in 53 cases (63.1%; 18.4% of the cohort), and any carbapenemase gene (KPC in 24 cases, NDM in 13 cases, IMP in 7 cases, VIM in 6 cases, and OXA-48-like in 1 case; not mutually exclusive) in 34 cases (40.5%; 11.8% of the cohort). Thirty-three patients (39.3% of gene-positive results) had more than one resistance gene category detected, consistent with polymicrobial or multi-target panel results.

### 2.5. Multivariable Analysis

**Figure 2:**
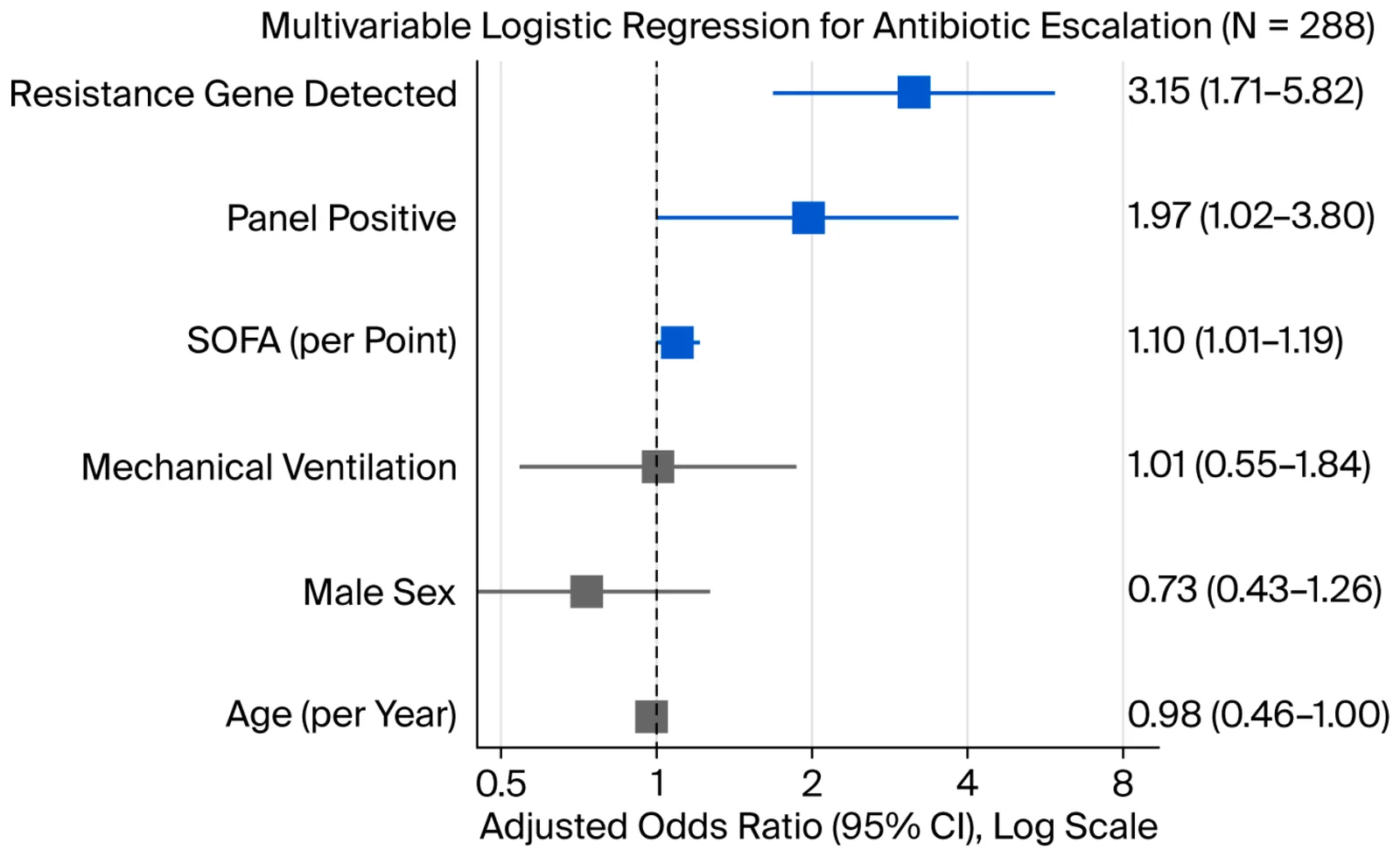
Multivariable logistic regression for antibiotic escalation (N = 288; covariates: sex, age, mechanical ventilation, SOFA, panel positivity, resistance gene detection), shown as adjusted odds ratios with 95% confidence intervals on a log scale. Resistance gene detection (aOR 3.15), panel positivity (aOR 1.97), and SOFA score (aOR 1.10 per point) were independent predictors; the dashed line marks the null (aOR = 1).

In the multivariable analysis, independent predictors of escalation were resistance gene detection (aOR 3.15, 95% CI 1.71–5.82, *p* < 0.001), panel positivity (aOR 1.97, 95% CI 1.02–3.80, *p* = 0.043), and SOFA score (aOR 1.10 per point, 95% CI 1.01–1.19, *p* = 0.026); sex, age, and mechanical ventilation were not independently associated (Table 3, Figure 2). In a sensitivity analysis excluding the collinear resistance gene term, panel positivity remained independently associated with escalation (aOR 3.13, 95% CI 1.72–5.70, *p* < 0.001), as did SOFA score (aOR 1.12, 95% CI 1.03–1.22, *p* = 0.005).

### 2.6. Antibiotic Regimens Before and After the Panel

**Figure 3:**
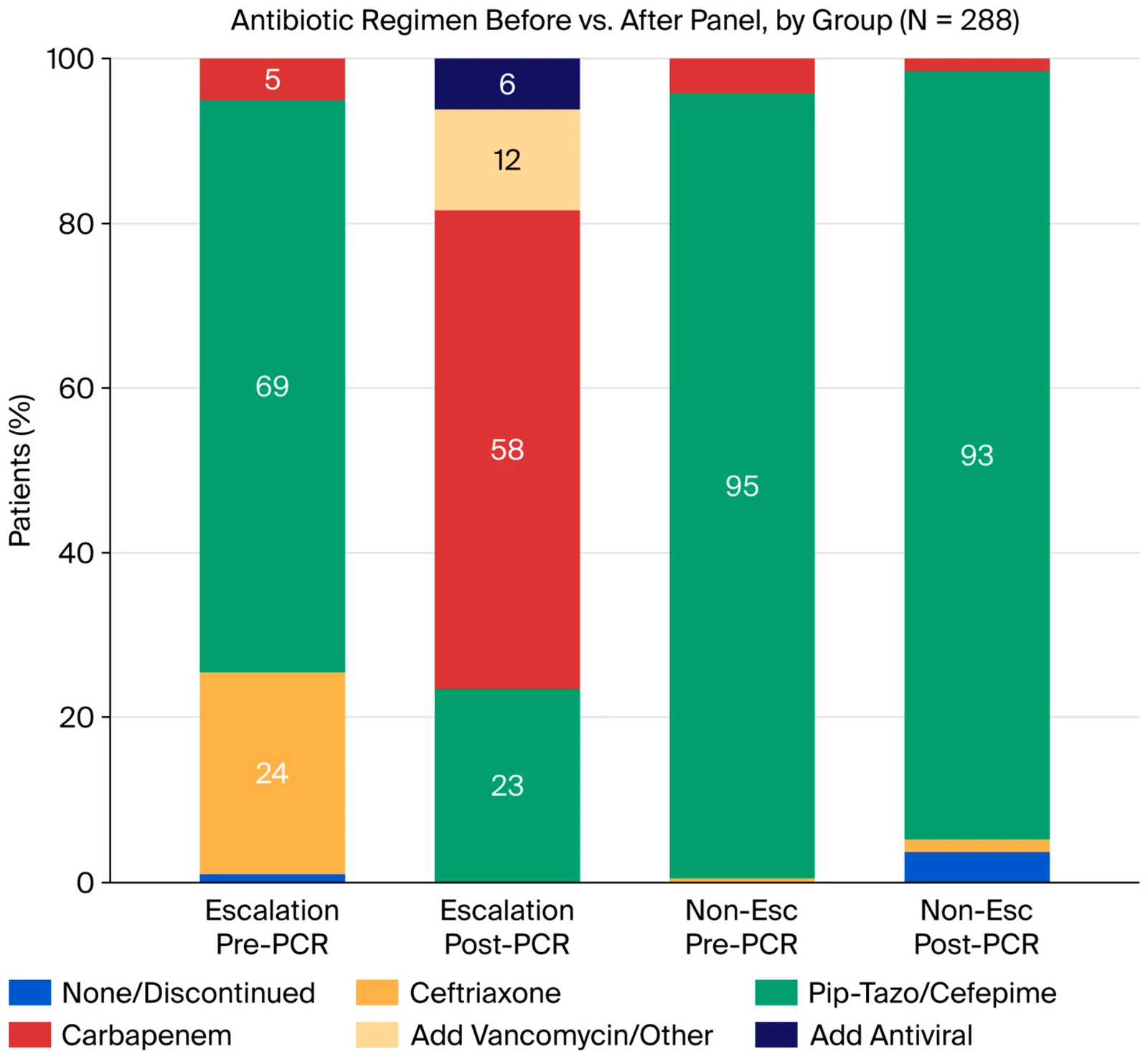
Antibiotic regimen composition before versus after the panel by group (100% stacked bars; escalation n = 98, non-escalation n = 190). In the escalation group, coverage shifted markedly toward carbapenems (and added vancomycin/antiviral) after the panel, whereas the non-escalation group remained on broad β-lactam therapy. Pip-tazo: piperacillin/tazobactam; PCR: Polymerase Chain Reaction of BioFire FilmArray Pneumonia Panel.

Before the panel, patients received a piperacillin/tazobactam- or cefepime-based regimen as the initial antibiotic in 249/288 cases (86.5%), a third-generation cephalosporin-based regimen in 25/288 cases (8.7%), and a carbapenem in 13/288 cases (4.5%). Patients in the escalation group were most often on a piperacillin–tazobactam- or cefepime-based regimen (69.4%), or a third-generation cephalosporin-based regimen (24.5%), with only 5.1% receiving a carbapenem. After the panel, carbapenem use rose to 58.2%, with vancomycin or another agent added in 12.2% of cases and an antiviral in 6.1% of cases. In the non-escalation group, broad empirical coverage (i.e., a piperacillin–tazobactam- or cefepime-based regimen) predominated both before (95.3%) and after (93.2%) the panel, with little change. Overall, carbapenem use increased from 4.5% before the panel to 20.8% after the panel (Figure 3). Patient-level regimen transitions are shown in Supplementary Figure S1.

Among the 57 patients escalated to a carbapenem, 17 (29.8%) had a carbapenemase gene and/or a carbapenem-resistant organism detected by panel/culture, a further 25 (43.9%) had some Gram-negative organism without a confirmed carbapenem resistance marker identified by panel or culture, and the remaining 15 (26.3%) had no organism identified by either method. Among the 12 patients who received an added anti-MRSA agent (vancomycin/teicoplanin), 8 (66.7%) had mecA/mecC/MREJ detected by the panel and/or *Staphylococcus aureus* recovered by culture or panel, while the remaining 4 (33.3%) had no *S. aureus*/mecA evidence according to either method. This descriptive concordance analysis does not constitute a clinical appropriateness adjudication (see Section Limitations).

### 2.7. Secondary Outcomes

Thirty-day mortality was 26.0% overall (75/288) and did not differ between groups (27.6% escalation vs. 25.3% non-escalation, *p* = 0.78). In-hospital mortality was 41.8% (escalation) versus 36.8% (non-escalation) (*p* = 0.49), and overall in-hospital mortality was 38.5% (111/288). Hospital LOS (median 25 vs. 23.5 days, *p* = 0.70), ICU LOS (11 vs. 11 days, *p* = 0.81), and ventilator duration (8 vs. 7 days, *p* = 0.29) did not differ (Table 4).

### 2.8. Survival and Mortality Models

**Figure 4:**
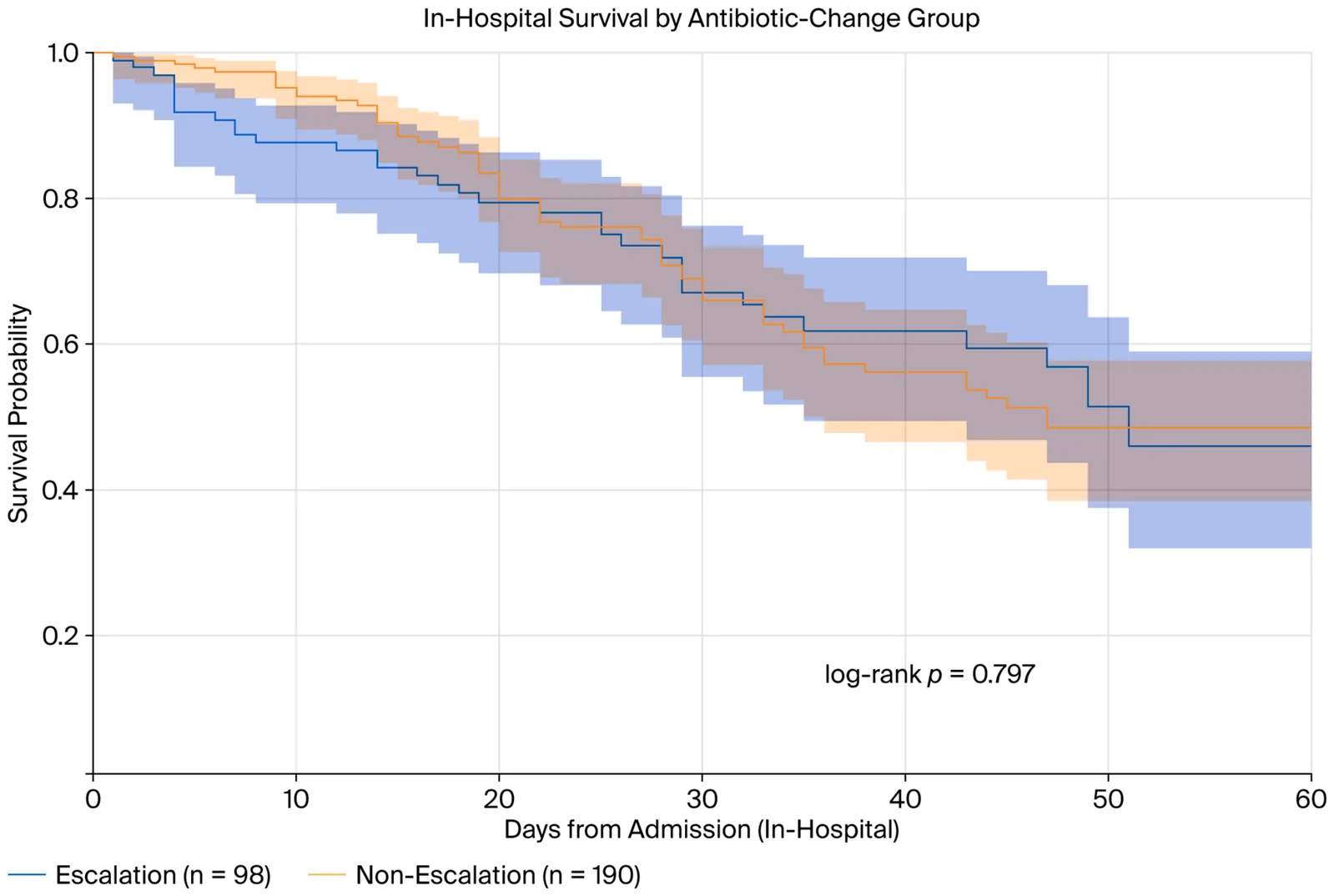
Kaplan–Meier in-hospital survival curves (time from admission, censored at discharge) for the escalation (n = 98) and non-escalation (n = 190) groups. Survival did not differ (log-rank *p* = 0.80).

In-hospital survival did not differ between escalation and non-escalation groups (Kaplan–Meier, log-rank *p* = 0.80; Figure 4). In a Cox proportional-hazards model for in-hospital mortality including escalation, sex, age, mechanical ventilation, SOFA, panel positivity, and resistance gene detection, escalation was not associated with mortality (aHR 1.12, 95% CI 0.74–1.70, *p* = 0.59). The only independent predictors of death were older age (aHR 1.02 per year, 95% CI 1.00–1.04, *p* = 0.032) and a higher SOFA score (aHR 1.08, 95% CI 1.02–1.15, *p* = 0.013), whereas panel positivity (aHR 0.89) and resistance gene detection (aHR 0.94) were not associated with mortality (Table 5).

### 2.9. Concordance with Sputum Culture

Sputum culture was positive in 61/288 cases (21.2%) (Table 1). Using culture as the reference standard and restricting the panel to bacterial targets, the panel had a sensitivity of 90.2% (55/61), a specificity of 57.7% (131/227), an overall concordance of 64.6% (186/288), a positive predictive value of 36.4%, and a negative predictive value of 95.6%. Among the 56 patients marked as positive by both methods, the detected organism agreed at the genus level in 52 (92.9%). A genus-level cross-tabulation of panel versus culture among bacteria-confirmed specimens (n = 157) is provided in Supplementary Table S2 (Table 6). Pathogen identification details are shown in Supplementary Tables S3 and S4.

### 2.10. Viral and Atypical Pathogen Detection

A respiratory virus was detected in 64/288 patients (22.2%; Influenza A: 15 cases, Coronavirus: 12 cases, Human Rhinovirus/Enterovirus: 12 cases, Respiratory Syncytial Virus: 12 cases, Human Metapneumovirus: 10 cases, Parainfluenza virus: 8 cases, Adenovirus: 4 cases) and an atypical bacterium in 2/288 (0.7%; *Legionella pneumophila*: 1 case, *Mycoplasma pneumoniae*: 1 case). Of the 64 virus-positive patients, 33 (51.6%) also had a bacterial target co-detected. An antiviral agent was added within 24 h of the panel result in 6/288 patients (2.1%), all six of whom were virus-positive (6/64, 9.4%) and none of which were virus-negative (0/224), indicating that antiviral escalation in this cohort was directly and exclusively linked to panel viral detection. These pathogens cannot be assessed by sputum culture and therefore fall outside the bacterial target concordance analysis in Section 2.9; their principal clinical contribution in this cohort was to inform antiviral, rather than antibacterial, decisions.

## 3. Discussion

This study shows that, in severe pneumonia requiring intensive care, although the antibiotic escalation group was in a more severe condition with a higher SOFA score compared to the non-escalation group, antibiotic escalation associated with rapid multiplex PCR testing was not accompanied by a statistically measurable difference in clinical outcome compared to non-escalation, within the power constraints of this retrospective cohort. Positive results from the panel or resistance gene tests were associated with antibiotic escalation.

In this ICU cohort with severe pneumonia, a rapid multiplex PCR panel was followed by antibiotic escalation in about one third of patients, an order of magnitude more often than de-escalation (34.0% vs. 2.8%). This pattern contrasts with the prevailing framing of these panels as primarily de-escalation tools [8,9,10,11]. For instance, in previous implementation studies centered on de-escalation, escalation typically occurred in roughly 8–10% of patients [12], far below the 34% observed here. A likely contributor to this difference is our institution’s empirical therapy pattern: a carbapenem was the initial empirical agent in only 4.5% (13/288) of patients, with most started on piperacillin–tazobactam or cefepime (86.5%) or ceftriaxone (8.7%); carbapenem use then rose to 20.8% after the panel (Supplementary Figure S1). Where baseline empirical coverage is already broad, for example, where initial carbapenem use is high, there is little room left to escalate, whereas our comparatively narrow empirical regimens left substantial headroom. This difference in baseline prescribing may partly explain why escalation was more frequent in our cohort than in panel studies framed around de-escalation, although baseline regimens were not directly compared across studies. The very low de-escalation rate likely also reflects the high illness severity and resistance burden of this CAP-ICU population, in whom clinicians may be reluctant to narrow therapy. We hypothesize that, in this setting, the panel functions chiefly to enable the early correction of inadequate empirical therapy rather than to reduce broad-spectrum exposure. In other words, the stepwise application of antibiotics based on rapid multiplex PCR is expected to reduce the indiscriminate overuse of carbapenems.

After adjustment for severity, escalation was independently associated with panel positivity and, most strongly, with resistance gene detection, a finding which is clinically coherent. Rapid identification of a pathogen, especially a resistance determinant, points clinicians toward the broader antibiotic coverage that culture would otherwise reveal only days later. Consistent with this, a randomized trial found that panel use shortened the time to antibiotic escalation against both Gram-positive and Gram-negative organisms [13]. Of the 84 resistance gene-positive results, methicillin resistance markers (mecA/mecC/MREJ) accounted for 36 (42.9%), CTX-M for 53 (63.1%), and any carbapenemase gene (KPC, NDM, IMP, VIM, OXA-48-like) for 34 (40.5%), with 33 patients testing positive for more than one category, consistent with polymicrobial or multi-target results. Because our institution’s multidrug resistance prevalence is high, the escalation rate observed here may not translate directly to settings with lower resistance prevalence, where a positive panel or resistance gene result would less often signal a truly drug-resistant organism and the yield of escalation would be expected to be correspondingly lower.

Escalation occurred in more severely ill patients, i.e., those with higher SOFA scores, yet it was not associated with worse 30-day mortality, in-hospital mortality, length of stay, or ventilator duration. This fits with prior work on ventilated ICU pneumonia: in one study, panel-derived information did not reliably predict clinical outcomes during treatment [14]; in another, panel use was not associated with 30-day mortality, antibiotic duration, or length of stay in a critically ill cohort [15]. This lack of association persisted in our survival analysis: escalation was unrelated to in-hospital survival in the Kaplan–Meier test (log-rank *p* = 0.80; Figure 4) and in an adjusted Cox model (aHR 1.12, 95% CI 0.74–1.70), and neither panel positivity nor resistance gene detection predicted mortality—only age and SOFA did (Table 5). Although still underpowered for mortality, the absence of an outcome penalty, even after accounting for severity, is consistent with escalation closing the gap left by inadequate empirical therapy rather than marking futile care. Notably, in the cohort in the previously mentioned study by Riaño-Sánchez et al., it was infectious diseases team involvement, rather than the test alone, that was associated with lower mortality [15], underscoring that the panel informs but does not replace expert interpretation.

We interpret the null association between panel positivity/resistance gene detection and mortality as reflecting three factors: (1) the cohort was not powered for a mortality difference (111 deaths, wide confidence intervals around all hazard ratios); (2) escalation itself may mediate and offset any risk conferred by a positive result, since clinicians acted on these findings; and (3) in this severely ill population, mortality is driven predominantly by host factors (age, SOFA score) rather than by the causative organism or the antibiotic regimen selected in response to it. This comparison was neither designed nor powered to demonstrate non-inferiority, and residual confounding is likely, since escalation was more common among patients with higher SOFA scores and greater microbiological risk, both of which independently predict mortality (Table 5). The absence of a measurable outcome difference should therefore be read as an absence of evidence of harm or benefit, not as evidence that escalation is clinically neutral.

Compared with sputum culture, the panel showed high sensitivity (90.2%) and negative predictive value (95.6%) with good genus-level agreement (92.9%) but lower specificity (57.7%). This may, in part, reflect the panel detecting organisms that culture did not recover—often where prior antibiotics suppressed growth—although true false-positive detection cannot be excluded without an independent reference standard. A negative panel did not exclude infection; that is, in culture-positive ventilator-associated pneumonia, non-targeted organisms occurred in 23% of cases, so the panel is not reliable enough to rule out bacterial infection and may warrant continued or broadened therapy [16]. Panel results should therefore be interpreted alongside clinical judgment and culture.

The 96 panel-positive/culture-negative results (Table 6) are clinically ambiguous: some plausibly represent organisms suppressed by prior antibiotics and missed by culture, but we cannot exclude that some represent detection of non-viable organisms, colonization, or low-level carriage without true infection. Because resistance gene detection was the strongest independent predictor of escalation (Table 3), any such discordance carries a direct risk of escalating therapy—particularly to carbapenems or anti-MRSA agents—without a confirmed bacterial infection driving it. Furthermore, because resistance genes are reported only alongside a corresponding bacterial call (Section 4.4), a resistance gene-positive result does not itself confirm active infection with a resistant organism at a clinically significant burden, and a low semi-quantitative bacterial load or a culture-negative counterpart cannot be distinguished from higher-burden, culture-confirmed cases in our dataset. A portion of the resistance gene-driven escalations may therefore reflect colonization or low-burden molecular detection rather than true resistant infection requiring the broadening of treatment—a possibility our data cannot exclude and that likely contributes to the modest positive predictive value observed against culture (Table 6). Consistent with this uncertainty, a descriptive concordance analysis of escalation decisions (Section 2.6) found that 26.3% of carbapenem escalations and 33.3% of anti-MRSA additions occurred without a corresponding resistance gene or organism finding stemming from either method. We could not adjudicate these as clinically appropriate or unnecessary without independent chart-level review, and we were similarly unable to adjudicate panel–culture discordant cases against an independent clinical reference standard. We flag both issues as priorities for prospective work.

Diagnostic speed alone does not guarantee appropriate action: translating a rapid panel result into a correct escalation, or non-escalation, decision requires stewardship interpretation, awareness of local resistance epidemiology, timely prescriber feedback, and, increasingly, structured decision support pathways [17]. Recent work on artificial intelligence- and machine learning-assisted stewardship decision support illustrates both the promise and the current external validation gaps of such tools relative to traditional risk scoring [18], underscoring that our panel’s value depends on the stewardship workflow surrounding it rather than the test result in isolation.

Our cohort’s age (median 72 years) and disease severity (median SOFA 8, shock in 49.0%, mechanical ventilation in 56.9%) place it within a population where comorbidity, frailty, and competing risks of death complicate the interpretation of any diagnostic or predictive tool, rapid molecular panels included [19]. Future implementation studies in similarly elderly, critically ill populations should account for these competing risks when evaluating panel-guided escalation.

### Limitations

Several limitations apply: The single-center, retrospective design lacks a culture-only comparator, so it cannot establish that the panel itself caused antibiotic changes, and the temporal association may be confounded by clinical deterioration. Because the panel was ordered at the discretion of treating clinicians, indication bias in who was tested is likely and limits generalizability. Resistance gene results were available only when a bacterial target was detected, which constrains and may bias that association. We addressed the resulting collinearity with a sensitivity analysis, but some resistance gene-driven escalations may still reflect colonization or low-burden molecular detection rather than confirmed resistant infection (see Section 3). We also could not analyze the proportion of patients on appropriate empirical therapy because of high missingness; without this indicator, we cannot distinguish escalation that corrected genuinely inadequate empirical coverage from escalation that broadened already-adequate therapy, which is why our central interpretation—that the panel chiefly enables correction of inadequate empirical therapy—remains a hypothesis rather than a demonstrated mechanism. Our institution requires antibiotic review board approval for carbapenem, vancomycin, and teicoplanin use (Section 4.3), and this administrative gatekeeping likely shapes both prescribing thresholds and the observed escalation rate, so results may not generalize to hospitals without a comparable antimicrobial approval system. We could not perform a clinician-adjudicated appropriateness review of individual escalation decisions; the descriptive concordance analysis in Section 2.6 is not a substitute for such review and does not establish clinical necessity. Finally, the secondary outcome comparisons were unadjusted and underpowered, particularly for mortality, and residual confounding cannot be excluded, since escalation was more common among patients with higher SOFA scores and greater microbiological risk.

## 4. Materials and Methods

### 4.1. Study Design and Setting

This retrospective study, conducted between April 2023 and September 2024, examined whether use of the BFPP (BioFire Diagnostics, LLC, Salt Lake City, UT, USA) affected antibiotic prescribing in patients admitted to the ICU with severe pneumonia. A total of 288 respiratory samples (sputum, tracheal aspirates, and BAL) were tested using both conventional culture and the BFPP. These tests were performed within 24 h of severe pneumonia detection.

The study protocol was approved by the Institutional Review Board of Daegu Catholic University Medical Center (IRB No. 2026-07-016).

### 4.2. Participants

The inclusion criteria were (1) age ≥ 18 years, (2) presence of clinical and radiological evidence for pneumonia according to the IDSA guidelines: new lung infiltrate on a chest X-ray and evidence that the infiltrate was of an infectious origin, i.e., possessing at least two of three clinical features (fever greater than 38 °C, leukocytosis or leukopenia, and purulent secretions), (3) BFPP performed, and (4) severe community-acquired pneumonia (CAP) presented. Severe pneumonia is most commonly defined using the ATS/IDSA (American Thoracic Society/Infectious Diseases Society of America) criteria, and this definition is used primarily to predict the need for ICU admission. Under this framework, a patient is classified as having severe CAP when they possess at least one of the following major criteria [20]: (1) septic shock requiring vasopressors and/or (2) respiratory failure requiring mechanical ventilation. A patient meeting three or more of the following (in the absence of a major criterion) is also considered to have severe pneumonia: respiratory rate ≥ 30 breaths/min, PaO_2_/FiO_2_ ratio ≤ 250, multilobar infiltrates, confusion or disorientation, uremia (BUN ≥ 20 mg/dL), leukopenia (WBC < 4000 cells/μL), thrombocytopenia (platelet count < 100,000/μL), hypothermia (core temperature < 36 °C), and hypotension requiring aggressive fluid resuscitation.

Patients with pneumonia onset ≥ 48 h after hospital admission or ventilator-associated pneumonia (onset ≥ 48 h after intubation) were excluded. Consistent with the 2019 ATS/IDSA guideline, which no longer designates healthcare-associated pneumonia as a separate category [21], community-onset pneumonia in patients transferred from long-term care facilities or other hospitals was classified as CAP. This population is reflected in the high comorbidity and multidrug resistance burden described in the Introduction and is discussed as a limitation to generalizability in the Section 3.

### 4.3. Variables and Definitions

This hospital has an antibiotic review board that oversees the use of antibiotics for infectious diseases, and physicians must obtain approval from this board to use antibiotics against MDR pathogens such as carbapenem, vancomycin, or teicoplanin. Therefore, in cases of severe pneumonia requiring ICU admission at this hospital, the initial antibiotic regimen is usually piperacillin/tazobactam + levofloxacin or moxifloxacin, or cefepime + levofloxacin or moxifloxacin [21]. ‘Escalation’ was defined as ‘switching to drugs effective against multidrug-resistant pathogens’ or ‘adding different-spectrum antibiotics or antiviral agents’ within 24 h of the panel result. At this hospital specifically, escalation refers to changing piperacillin/tazobactam or cefepime to a carbapenem, or adding vancomycin or teicoplanin to the antibiotic originally used. Escalation also includes changing ceftriaxone to piperacillin/tazobactam or cefepime or adding an antiviral agent. ‘De-escalation’ was defined as discontinuation of antibiotics and/or switching of the previous antibiotic to a narrower-spectrum antibiotic within 24 h of the panel result, for example, discontinuing the administration of moxifloxacin or levofloxacin or changing piperacillin/tazobactam or cefepime to ceftriaxone or a narrower-range antibiotic. Thirty-day mortality was defined as death within 30 days of hospital admission.

#### 4.3.1. Data Collection

Clinical and demographic data were retrospectively obtained from the medical records. We collected demographic data such as age and gender, and clinical data such as admission date, ICU admission date, discharge date, death status, severity scores, comorbidities, vital signs, use of ventilator, results of conventional culture and BFPP, and antibiotics prescribed.

#### 4.3.2. Endpoints

The primary endpoint was the number of antibiotic escalations according to BFPP results and associated factors. Secondary outcomes were 30-day mortality, in-hospital mortality, ICU length of stay, and duration of mechanical ventilation according to antibiotic escalation and non-escalation status.

### 4.4. Measurement

The BFPP (BioFire Diagnostics, LLC, Salt Lake City, UT, USA) was used for the simultaneous detection and identification of respiratory pathogens and antimicrobial resistance genes in lower respiratory tract specimens. The panel is a closed, sample-to-answer system that integrates nucleic acid extraction, reverse transcription, and nested multiplex PCR amplification followed by melting curve analysis for target detection within approximately one hour (about 75 min, with less than 5 min of hands-on time). The assay detects 33 targets, comprising 18 bacteria (15 typical bacteria reported semi-quantitatively as genome copies per milliliter [10^4^ to ≥10^7^ copies/mL], and 3 atypical bacteria—*Legionella pneumophila*, *Mycoplasma pneumoniae*, and *Chlamydia pneumoniae*), 8 respiratory viruses, and 7 antimicrobial resistance genes (1 for methicillin resistance [mecA/mecC and MREJ], 1 for extended-spectrum β-lactamases [CTX-M], and 5 for carbapenem resistance [KPC, NDM, OXA-48-like, VIM, and IMP]) [22,23].

Testing was performed according to the manufacturer’s instructions. Briefly, the reagent pouch was hydrated with the hydration solution, the specimen was mixed with sample buffer and loaded into the pouch via the provided injection vials, and the pouch was inserted into the BioFire^®^ FILMARRAY^®^ [2.0/TORCH] instrument (BioFire Diagnostics, LLC, Salt Lake City, UT, USA). All subsequent steps, including extraction, amplification, and result interpretation, were carried out automatically by the system, and results were reported as “detected” or “not detected” for each target, with semi-quantitative bin values reported for the 15 typical bacterial targets.

Sputum conventional culture was also performed as soon as pneumonia was detected in all cases.

By design, the panel reports its seven resistance gene targets only in association with detection of a corresponding bacterial organism (e.g., mecA/mecC/MREJ is reported only when *Staphylococcus aureus* is detected, CTX-M only with a relevant Gram-negative target, and carbapenemase genes only with a corresponding Gram-negative target); resistance genes are not reported as organism-independent findings. This technical characteristic constrains interpretation of resistance gene-positive results (see Section Limitations).

### 4.5. Statistical Analysis

All data were anonymously collected and stored in a secure database. Normally distributed continuous variables were expressed as means; non-normally distributed continuous variables were expressed as medians and interquartile ranges (IQRs); categorical variables were expressed as numbers and percentages. Quantitative results in the ‘escalation’ and ‘non-escalation’ groups were compared using the Mann–Whitney U test. Qualitative variables between the two groups (escalation vs. non-escalation) were compared using chi-square tests. To identify the factors affecting the selection of antibiotics, univariate and multivariable logistic regression models were used and expressed as odds ratios (ORs) and 95% confidence intervals (CIs). In-hospital survival (time from admission to death, censored at discharge) was compared between escalation and non-escalation groups using Kaplan–Meier curves with the log-rank test. Predictors of in-hospital mortality were examined using univariable and multivariable Cox proportional-hazards regression including escalation, sex, age, mechanical ventilation, SOFA, panel positivity, and resistance gene detection. As an exploratory secondary analysis, sputum culture—obtained in all patients—served as the reference standard for panel diagnostic performance (sensitivity, specificity, overall concordance, predictive values, and organism-level [genus] agreement), restricted to bacterial panel targets for comparability with culture. A two-sided *p* < 0.05 was considered significant. All statistical analyses were performed using IBM SPSS Statistics version 25 (IBM Corp., Armonk, NY, USA) and Python 3 (statsmodels/scipy).

## 5. Conclusions

In severe pneumonia requiring intensive care and managed with a comparatively narrow initial empirical regimen, antibiotic escalation, rather than de-escalation, was the predominant change following rapid multiplex PCR panel testing and was independently associated with panel positivity and resistance gene detection. No statistically measurable difference in clinical outcomes was observed; however, the study was not powered to exclude any clinically meaningful benefit or harm of escalation, and this finding should not be read as evidence that escalation is clinically inconsequential. Clinicians caring for critically ill patients with severe pneumonia—particularly at institutions with similarly narrow empirical regimens and antimicrobial approval systems—should anticipate that a positive panel, particularly with a resistance gene, may prompt early broadening of therapy; however, prospective, adequately powered studies that also adjudicate escalation appropriateness are needed to confirm whether panel-guided escalation improves outcomes.

## Figures and Tables

**Table 1:** Baseline characteristics by escalation status.

	Escalation (N = 98)	Non-Escalation (N = 190)	All Subjects (N = 288)	*p*
Age, years	71 (62–81)	72 (63–79)	72 (63–80)	0.594
Sex, male	56 (57.1)	119 (62.6)	175 (60.8)	0.437
Comorbidity				
Hypertension	40 (41.7)	82 (44.1)	122 (42.4)	0.794
Neurologic diseases	43 (43.9)	54 (28.7)	97 (33.7)	0.015 *
Diabetes mellitus	31 (31.6)	58 (31.2)	89 (30.9)	1.000
Chronic pulmonary conditions ^a^	26 (26.5)	59 (31.1)	85 (29.5)	0.509
Heart diseases	25 (26.0)	46 (24.2)	71 (24.7)	0.847
Immune suppression ^b^	18 (18.4)	41 (21.6)	59 (20.5)	0.627
Malignancy	17 (17.3)	26 (13.7)	43 (14.9)	0.514
Chronic renal disease	12 (12.4)	21 (11.1)	33 (11.5)	0.892
Chronic liver disease	8 (8.2)	9 (4.7)	17 (5.9)	0.365
Endocrine illness	10 (10.2)	3 (1.6)	13 (4.5)	0.002 *
SOFA score at the time of sampling	9 (6–11)	7 (4–9)	8 (5–10)	0.001 *
APACHE-II	19 (15–24)	18 (14–23)	19 (14–24)	0.165
Shock	52 (53.1)	89 (46.8)	141 (49.0)	0.381
Ventilator use	62 (63.3)	102 (53.7)	164 (56.9)	0.153
Microbiological sampling site				0.208
Endotracheal aspiration	62 (63.3)	100 (52.6)	162 (56.3)	
Sputum	35 (35.7)	86 (45.3)	121 (42.0)	
Bronchoalveolar lavage	1 (1.0)	4 (2.1)	5 (1.7)	
Positive result of rapid multiplex PCR	80 (81.6)	111 (58.4)	191 (66.3)	<0.001 *
Resistance gene detected	48 (49.0)	36 (18.9)	84 (29.2)	<0.001 *
Positive result of sputum culture	36 (36.7)	25 (13.2)	61 (21.2)	<0.001 *
Results turnaround, hours				
Rapid multiplex PCR	2.0 (1.7–3.0)	2.1 (1.6–3.0)	2.0 (1.6–3.0)	0.509
Sputum culture	70.5 (53–88)	65.0 (53–108)	67.0 (52.5–94)	0.775

SOFA: Sequential Organ Failure Assessment; APATCHE-II: Acute Physiology And Chronic Health Evaluation II; PCR: Polymerase Chain Reaction. ^a^ Chronic pulmonary condition includes patients with a history of chronic obstructive pulmonary disease, severe bronchiectasis, tuberculosis or infection-related destroyed lung, and chronic respiratory insufficiency. ^b^ Immunosuppression is defined as leucopenia < 1 Giga/L, HIV infection, history of solid organ or stem cell transplantation, and immunosuppressive treatment including corticosteroids over 10 mg/day of equivalent prednisone for more than 15 days. Data are median (interquartile range) or N (%). * Chi-square test, *p* < 0.05.

**Table 2:** Antibiotic escalation by exposure.

Exposure	Escalation, n/Panel Results, N (%)	*p*
Panel positive	80/191 (41.9)	<0.001 *
Panel negative	18/97 (18.6)	
Resistance gene detected	48/84 (57.1)	<0.001 *
No resistance gene	50/204 (24.5)	

* Chi-square test, *p* < 0.05.

**Table 3:** Univariable and multivariable logistic regression for antibiotic escalation (N = 288, events = 98).

Variable	Unadjusted OR (95% CI)	*p*	Adjusted OR (95% CI)	*p*
Male sex	0.80 (0.48–1.31)	0.366	0.73 (0.43–1.26)	0.260
Age (per year)	0.99 (0.97–1.01)	0.317	0.98 (0.96–1.00)	0.060
Mechanical ventilation	1.49 (0.90–2.45)	0.121	1.01 (0.55–1.84)	0.981
SOFA (per point)	1.12 (1.04–1.20)	0.001 *	1.10 (1.01–1.19)	0.026 *
Panel positive	3.16 (1.76–5.69)	<0.001 *	1.97 (1.02–3.80)	0.043 *
Resistance gene detected	4.11 (2.40–7.03)	<0.001 *	3.15 (1.71–5.82)	<0.001 *

OR: Odds Ratio; SOFA: Sequential Organ Failure Assessment. Covariates: sex, age, mechanical ventilation, SOFA, panel positivity, resistance gene detection. * *p* < 0.05.

**Table 4:** Secondary outcomes by escalation status.

Outcome	Escalation (N = 98)	Non-Escalation (N = 190)	All Subjects (N = 288)	*p*
30-day mortality	27 (27.6)	48 (25.3)	75 (26.0)	0.781
In-hospital mortality	41 (41.8)	70 (36.8)	111 (38.5)	0.486
Hospital LOS, median, days	25 (14–46)	23.5 (14–42)	25 (14–43)	0.703
ICU LOS, days	11 (6–20)	11 (6–19)	11 (6–20)	0.807
Ventilator duration, days	8 (4–22)	7 (4–14)	8 (4–17)	0.286

LOS: Length Of Stay; ICU: Intensive Care Unit. Data are N (%) or median (IQR).

**Table 5:** Univariable and multivariable Cox proportional-hazards analysis for in-hospital mortality (N = 288, deaths = 111).

Variable	Unadjusted HR (95% CI)	*p*	Adjusted HR (95% CI)	*p*
Escalation	1.05 (0.71–1.54)	0.808	1.12 (0.74–1.70)	0.590
Male sex	1.03 (0.70–1.51)	0.893	1.02 (0.69–1.51)	0.925
Age (per year)	1.02 (1.00–1.04)	0.032	1.02 (1.00–1.04)	0.032
Mechanical ventilation	1.03 (0.71–1.50)	0.884	0.83 (0.56–1.25)	0.379
SOFA score (per point)	1.07 (1.01–1.13)	0.019	1.08 (1.02–1.15)	0.013
Panel positive	0.90 (0.61–1.33)	0.602	0.89 (0.56–1.40)	0.613
Resistance gene detected	1.06 (0.71–1.57)	0.783	0.94 (0.58–1.52)	0.794

HR: Hazard Ratio; SOFA: Sequential Organ Failure Assessment. Cox proportional-hazards model (in-hospital death, censored at discharge). Covariates: escalation, sex, age, mechanical ventilation, SOFA, panel positivity, resistance gene detection. Deaths = 111/288.

**Table 6:** Diagnostic performance of the multiplex PCR panel versus sputum culture (reference standard; bacterial targets; N = 288, culture-positive = 61).

Metric	Value, n (%)
Sensitivity	55/61 (90.2)
Specificity	131/227 (57.7)
Overall concordance	186/288 (64.6)
Positive predictive value	55/151 (36.4)
Negative predictive value	131/137 (95.6)
Organism agreement at genus level (both positive, n = 56)	52/56 (92.9)

2 × 2 (bacterial targets): true positive: 55, false positive: 96, false negative: 6, true negative: 131. Lower specificity/PPV may in part reflect panel detection of organisms not recovered by culture (e.g., suppressed by prior antibiotics); however, true false-positive detection cannot be excluded without an independent clinical reference standard.

## Data Availability

The data presented in this study are available on request from the corresponding author. The data are not publicly available due to institutional and patient privacy restrictions.

## Supplementary Materials

The following supporting information can be downloaded at: https://www.mdpi.com/article/10.3390/antibiotics15090903/s1, Figure S1: Patient-level transitions of antibiotic regimen from before to after the multiplex PCR panel; Table S1: Clinical characteristics between escalation, deescalation and maintaining of antibiotics groups; Table S2: Genus-level cross-tabulation of panel-detected versus sputum culture organisms; Table S3: Pathogen identification from positive results of conventional sputum culture and rapid multiplex PCR tests; Table S4: Diagnostic performance of the multiplex PCR panel versus sputum culture.

## References

[1] Reyes L.F., Conway Morris A., Serrano-Mayorga C., Derde L.P.G., Dickson R.P., Martin-Loeches I. (2025). Community-Acquired Pneumonia. Lancet.

[2] Cilloniz C., Ferrer M., Liapikou A., Garcia-Vidal C., Gabarrus A., Ceccato A., de La Bellacasa J.P., Blasi F., Torres A. (2018). Acute Respiratory Distress Syndrome in Mechanically Ventilated Patients with Community-Acquired Pneumonia. Eur. Respir. J..

[3] Peiffer-Smadja N., Bouadma L., Mathy V., Allouche K., Patrier J., Reboul M., Montravers P., Timsit J.-F., Armand-Lefevre L. (2020). Performance and Impact of a Multiplex PCR in ICU Patients with Ventilator-Associated Pneumonia or Ventilated Hospital-Acquired Pneumonia. Crit. Care.

[4] Yoo N., Cheng X.J.C., Chung J., Chawla S., Zacharioudakis I., Dubrovskaya Y. (2025). Safety and Efficacy of Antimicrobial Optimization Based on Negative Results from BioFire FilmArray Pneumonia Panel and Respiratory Culture. Antimicrob. Steward. Healthc. Epidemiol..

[5] Alsayed A.R., Zihlif M., Abuata O.M., Permana A.D., Zihlif M. (2026). Molecular Diagnostics, Antimicrobial Resistance Patterns, and Clinical Outcomes in Hospitalized Pneumonia Patients: A Prospective Study from Jordan and a Call for National Guideline Integration. Infect. Drug Resist..

[6] Webber D.M., Wallace M.A., Burnham C.-A.D., Anderson N.W. (2020). Evaluation of the BioFire FilmArray Pneumonia Panel for Detection of Viral and Bacterial Pathogens in Lower Respiratory Tract Specimens in the Setting of a Tertiary Care Academic Medical Center. J. Clin. Microbiol..

[7] Kosai K., Akamatsu N., Ota K., Mitsumoto-Kaseida F., Sakamoto K., Hasegawa H., Izumikawa K., Mukae H., Yanagihara K. (2022). BioFire FilmArray Pneumonia Panel Enhances Detection of Pathogens and Antimicrobial Resistance in Lower Respiratory Tract Specimens. Ann. Clin. Microbiol. Antimicrob..

[8] Ibars R.L., Carvalho-Brugger S., Ibáñez P.R., Vidal M.V., Moles S.I., Torner M.M., Bellés A.B., Castellano A., Campi D., Caballero J. (2026). Evaluating the Impact of FilmArray Pneumonia Plus Panel in Therapeutic Decision-Making in Critical Patients with Suspected Respiratory Infection. Antibiotics.

[9] Hou C., Ali S., Vyas N. (2020). Impact of FilmArray Pneumonia Panel on Early Targeted Antibiotic Therapy. Infect. Control Hosp. Epidemiol..

[10] Cano S., Clari M.Á., Albert E., Liñan J., Juan M., Olea B., Carbonell N., Navarro D. (2026). Protocolized Antimicrobial Stewardship Following FilmArray Pneumonia Plus Panel Testing in Mechanically Ventilated Patients with Severe Lower Respiratory Tract Infection. Sci. Rep..

[11] Stafylaki D., Maraki S., Vaporidi K., Georgopoulos D., Kontoyiannis D.P., Kofteridis D.P., Chamilos G. (2022). Impact of Molecular Syndromic Diagnosis of Severe Pneumonia in the Management of Critically Ill Patients. Microbiol. Spectr..

[12] Miller M.M., Van Schooneveld T.C., Stohs E.J., Marcelin J.R., Alexander B.T., Watkins A.B., Creager H.M., Bergman S.J. (2023). Implementation of a Rapid Multiplex Polymerase Chain Reaction Pneumonia Panel and Subsequent Antibiotic De-Escalation. Open Forum Infect. Dis..

[13] Virk A., Strasburg A.P., Kies K.D., Donadio A.D., Mandrekar J., Harmsen W.S., Stevens R.W., Estes L.L., Tande A.J., Challener D.W. (2024). Rapid Multiplex PCR Panel for Pneumonia in Hospitalised Patients with Suspected Pneumonia in the USA: A Single-Centre, Open-Label, Pragmatic, Randomised Controlled Trial. Lancet Microbe.

[14] Dessajan J., Thy M., Doman M., Stern J., Gallet A., Fouque G., Chosidow S., Ruckly S., Gueye S., Lamara F. (2025). Assessing FilmArray Pneumonia+ Panel Dynamics During Antibiotic Treatment to Predict Clinical Success in ICU Patients with Ventilated Hospital-Acquired Pneumonia and Ventilator-Associated Pneumonia: A Multicenter Prospective Study. Crit. Care.

[15] Riaño-Sánchez L.F., Alvarez-Moreno C.A., Godoy M., Sierra C.R., Castañeda M.I., Cortés J.A. (2025). Multiplex PCR Pneumonia Panel in Critically Ill Patients Did Not Modify Mortality: A Cohort Study. Antibiotics.

[16] Berteau F., Kouatchet A., Le Gall Y., Pouplet C., Delbove A., Darreau C., Lemarie J., Jarousseau F., Reizine F., Giacardi C. (2025). Epidemiology and Prediction of Non-Targeted Bacteria by the FilmArray Pneumonia Plus Panel in Culture-Positive Ventilator-Associated Pneumonia: A Retrospective Multicentre Analysis. Ann. Intensive Care.

[17] Waldock W.J., Gilchrist M., Ashrafian H., Darzi A., Dean Franklin B. (2025). A Mixed Methods Evaluation of an Antimicrobial Prescribing Clinical Decision Support System App. npj Antimicrob. Resist..

[18] Pinto A., Pennisi F., Ricciardi G.E., Signorelli C., Gianfredi V. (2025). Evaluating the Impact of Artificial Intelligence in Antimicrobial Stewardship: A Comparative Meta-Analysis with Traditional Risk Scoring Systems. Infect. Dis. Now.

[19] Pinto A., Pennisi F., Odelli S., De Ponti E., Veronese N., Signorelli C., Baldo V., Gianfredi V. (2025). Artificial Intelligence in the Management of Infectious Diseases in Older Adults: Diagnostic, Prognostic, and Therapeutic Applications. Biomedicines.

[20] Mandell L.A., Wunderink R.G., Anzueto A., Bartlett J.G., Campbell G.D., Dean N.C., Dowell S.F., File T.M., Musher D.M., Niederman M.S. (2007). Infectious Diseases Society of America/American Thoracic Society Consensus Guidelines on the Management of Community-Acquired Pneumonia in Adults. Clin. Infect. Dis..

[21] Metlay J.P., Waterer G.W., Long A.C., Anzueto A., Brozek J., Crothers K., Cooley L.A., Dean N.C., Fine M.J., Flanders S.A. (2019). Diagnosis and Treatment of Adults with Community-Acquired Pneumonia. An Official Clinical Practice Guideline of the American Thoracic Society and Infectious Diseases Society of America. Am. J. Respir. Crit. Care Med..

[22] Murphy C.N., Fowler R., Balada-Llasat J.M., Carroll A., Stone H., Akerele O., Buchan B., Windham S., Hopp A., Ronen S. (2020). Multicenter Evaluation of the BioFire FilmArray Pneumonia/Pneumonia Plus Panel for Detection and Quantification of Agents of Lower Respiratory Tract Infection. J. Clin. Microbiol..

[23] Khan M.A., Bajwa A., Hussain S.T. (2025). Pneumonia: Recent Updates on Diagnosis and Treatment. Microorganisms.

