# Supplementary material for: Antibiotic Escalation Following Rapid Multiplex PCR Pneumonia Panel Testing in Intensive Care Patients with Severe Pneumonia: A Retrospective Cohort Study
Source: Antibiotics (Basel). 2026 Sep 14;15(9):903. doi: 10.3390/antibiotics15090903 (PMC13603844; doi:10.3390/antibiotics15090903)
Supplement: Supplementary file 1 [file antibiotics-15-00903-s001.zip › antibiotics-4551058-supplementary.pdf]

Supplementary Material

Impact of a Multiplex PCR Pneumonia Panel on Antibiotic Escalation in Severe CAP: A Retrospective Cohort Study

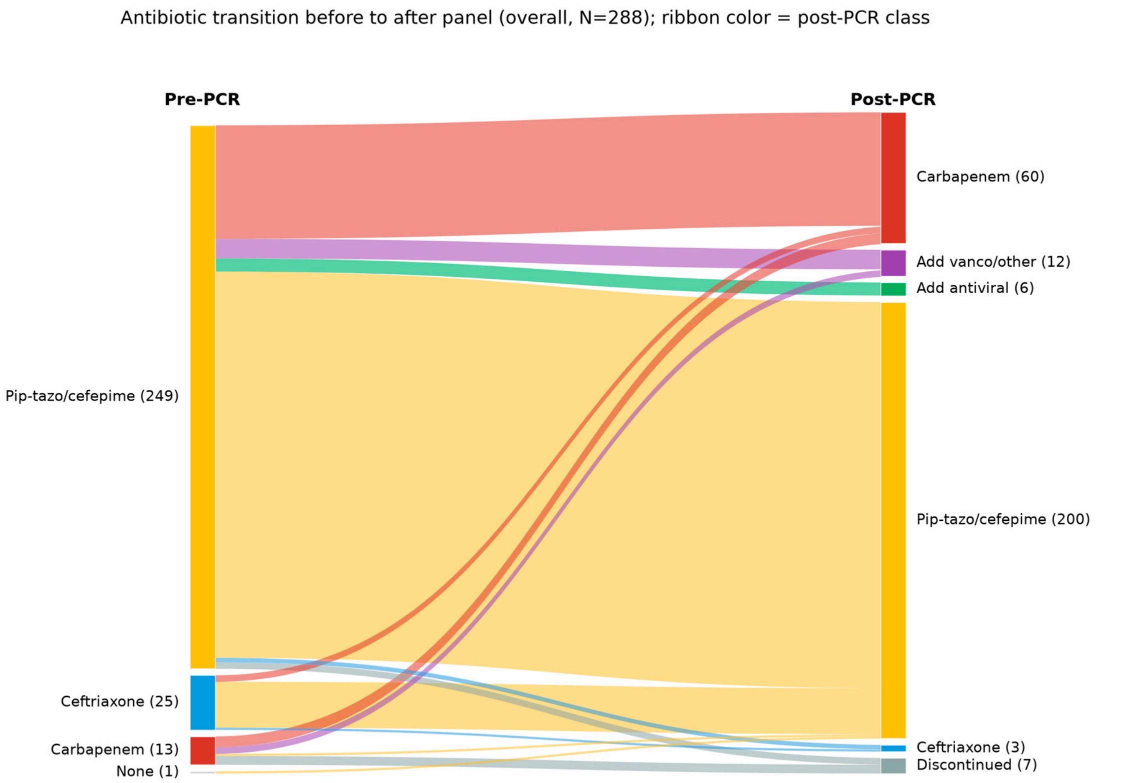

Supplementary Figure S1. Patient-level transitions of antibiotic regimen from before to after the multiplex PCR panel (alluvial/Sankey diagram; overall cohort, N=288). Left nodes show the pre-panel empirical regimen and right nodes the post-panel regimen; ribbon width is proportional to the number of patients and ribbon color denotes the post-panel antibiotic class. Flows converging on carbapenem (red) from piperacillin–tazobactam/cefepime and ceftriaxone illustrate the predominant escalation pathway.

Supplementary Table S1. Clinical characteristics between escalation, deescalation and maintaining of antibiotics groups.

	Escalation (N=98)	Deescalation (N=10)	Maintaining (N=180)	Total (N=288)	P
Ventilator	62 (37.8)	6 (3.7)	96 (58.5)	164 (100)	0.274
Sample types					<0.001*
Endotracheal aspiration	62 (38.3)	5 (3.1)	95 (58.6)	162 (100)	
Sputum	35 (28.9)	3 (2.5)	83 (68.6)	121 (100)	
BAL	1 (20.0)	2 (40.0)	2 (40.0)	5 (100)	
Positive result in rm-PCR	80 (41.9)	6 (3.1)	105 (55.0)	191 (100)	<0.001*
SOFA	9 (7-11)	5.5 (3-8)	7 (4-9)	8 (5-10)	0.003*
APACHE-II	19 (15-24)	19 (16.5-26.8)	18 (16-23)	19 (14-24)	0.220
Age	71 (62-81)	72 (65-75)	73 (63-79)	72 (63-80)	0.752
Duration of positive results in sputum culture, hours	69.0 (52.3-89.0)	54.0	66.0 (53.0-108.0)	67 (52-94)	0.775
Duration of positive results in rm-PCR, hours	2.0 (1.8-2.8)	1.8 (1.6-2.0)	2.0 (1.5-3.4)	2 (1.6-3)	0.309
30-day mortality	27 (27.6)	3 (30.0)	45 (25.0)	75 (26.0)	1.000
In-hospital mortality	41 (41.8)	6 (60.0)	64 (35.6)	111 (38.5)	0.627
Duration of admission, days	26 (15.5-46)	15.5 (11-25)	24 (14-41)	25 (14-43)	0.664
Duration of ICU admission, days	23 (11-40)	10 (5.5-16.5)	19 (11-33)	19 (11-34)	0.852
Duration of ventilator use, days	8 (4-22)	5 (3.5-11)	7.5 (4-14.8_	8 (4-17)	0.403

*p<0.05

Kruskal-Wallis test for continuous variables, chi-square test for categorical variables

Supplementary Table S2. Genus-level cross-tabulation of panel-detected (lower-respiratory) versus sputum-culture organisms

Bacteria-confirmed specimens only (panel bacterial-positive or culture-positive), n=157 of the total cohort N=288 (culture-positive 61, panel bacterial-positive 151). Panel+ = organism detected by panel; Culture+ = organism grown on sputum culture. Total = specimens positive by either method.

**Organism (genus)**	**Panel+ & Culture+**	**Panel+ only**	**Culture+ only**	**Total (either+)**	**Panel− & Culture−**
Acinetobacter	8	20	1	29	128
Klebsiella pneumoniae	13	49	3	65	92
Pseudomonas aeruginosa	15	19	2	36	121
Staphylococcus aureus	11	31	1	43	114
Escherichia coli	5	14	0	19	138
Enterobacter cloacae	0	6	3	9	148
Haemophilus influenzae	0	33	0	33	124
Serratia marcescens	2	1	0	3	154

Specimen-level (bacteria-confirmed n=157): panel-bacterial+/culture+ 55, panel+/culture− 96, panel−/culture+ 6. The low culture-only counts indicate the panel recovered most culture-positive organisms; the high panel-only counts reflect panel detection of organisms not grown by culture (e.g., suppressed by prior antibiotics).

Supplementary Table S3. Pathogen identification from positive results of conventional sputum culture and rapid multiplex PCR tests.

	Conventional sputum culture (N=61)	Rapid multiplex PCR (N=191)
Acinetobacter	8	28
K. pneumoniae	15	62
E. coli	5	19
Pseudomonas	16	34
Enterobacter	3	6
Citrobacter	1	-
Serratia	2	3
S. aureus	11	42
H. influenzae	-	33
M. catarrhalis	-	4
Proteus	-	7
S. pneumoniae	-	13
Legionella	-	1
Mycoplasma pneumoniae	-	1
Viruses		
adenovirus		4
Coronavirus		12
Human metapneumo		10
Human rhino/enterovirus		12
Influenza A		15
Parainfluenza		8
RSV		12
CTXM		53
		CPE in rm-PCR (N=34)
IMP		7
KPC		24
NDM		13
OXA-48 like		1
VIM		6
MecA/C		36
Sputum CRE	11	

N (%)

Supplementary Table S4. Diagnostic performance of the multiplex PCR panel versus sputum culture. (reference standard; bacterial targets; N=288, culture-positive 61)

Sputum Culture / RM-PCR	Acinetobacter baumannii-calceaceticus (N=8)	Klebsiella pneumonia (N=15)	Escherichia coli (N=5)	Pseudomonas aeruginosa (N=16)	Enterobacter cloacae complex (N=3)	Serratia marcescens (N=2)	Staphylococcus aureus (N=11)	P
Acinetobacter baumannii-calceaceticus (N=17)	7 (41.2)	5 (29.4)	1 (5.9)	3 (17.6)	0	1 (5.9)	0	0.003*
Klebsiella pneumonia (N=23)	4 (8.7)	12 (52.2)	3 (13.0)	5 (21.7)	0	0	1 (4.3)	0.004*
Escherichia coli (N=12)	1 (8.3)	4 (33.3)	5 (41.7)	2 (16.7)	0	0	0	0.001*
Pseudomonas aeruginosa (N=24)	2 (8.3)	6 (25.0)	0	15 (62.5)	1 (4.2)	0	0	<0.001**
Enterobacter cloacae complex (N=2)	0	1 (50.0)	1 (50.0)	0	0	0	0	0.501
Serratia marcescens (N=2)	0	0	0	0	0	2 (100.0)	0	<0.001**
Staphylococcus aureus (N=23)	4 (17.4)	4 (17.4)	1 (4.3)	4 (17.4)	0	0	10 (43.5)	0.006*

*<0.005 in Chi-square test, **<0.001 in Chi-square test
